# Mediating effect of the parent-child relationship on the association between parenting stress and children’s eating behaviors

**DOI:** 10.1186/s12889-021-12052-5

**Published:** 2021-10-30

**Authors:** Myoungock Jang, Roger Brown, Moonkyoung Park

**Affiliations:** 1 College of Nursing, Chungnam National University, Munhwa-ro 266, Jung-gu, Daejeon, 35015 South Korea; 2 School of Nursing, University of Wisconsin-Madison, Signe Skott Cooper Hall, 701 Highland Avenue, Madison, WI 53705 USA

**Keywords:** Parenting stress, Parent–child relationship, Child eating behaviors

## Abstract

**Background:**

Emerging evidence suggests that parenting stress plays a significant role in children’s eating behavior. However, the nature of the relationship between parenting stress and children’s health behaviors is still not well understood, possibly because there is limited understanding of the mediating factors. The purpose of this study was to examine the mediating effect of the parent–child relationship on the association between parenting stress and children’s eating behaviors in families with young children.

**Methods:**

Using a cross-sectional study design, we recruited mothers of families with children aged four to six years in the United States. We asked the mothers select one child if she has more than one eligible child. Mothers answered well-validated questionnaires regarding parenting stress, the parent–child relationship, and children’s eating behaviors. We utilized a structural equation model to analyze the mediating factors.

**Results:**

A total of 172 mothers of children participated in this study. The children’s mean age was 4.92 (SD 0.89) years; 50% of children were female and 71.2% were non-Hispanic Whites. Parenting stress was associated with subcategories of the parent–child relationship (satisfaction with parenting [b* = − 0.69, *p* < .01], communication [b* = 0.45, *p* < 0.01], and limit setting [b* = − 0.82, *p* < .01]). The subcategories of communication and limit setting were negatively associated with food responsiveness in children (b* = − 0.24, *p* < .01; b* = − 0.46, *p* < .01, respectively). Limit setting was negatively associated with emotional overeating in children (b* = − 0.49, *p* < .01). Communication mediated the association between parenting stress and food responsiveness in children (b* = − 0.11, *p* < .01). The mediating role of limit setting was established in the association between parenting stress and food responsiveness as well as in the association between parenting stress and emotional overeating (b* = 0.38, *p* < .01; b* = 0.40, *p* < .01, respectively).

**Conclusions:**

The parent–child relationship is an important component in improving children’s eating behaviors in families that have parents with higher parenting stress levels.

## Background

Childhood obesity is a major global health concern given the increased rate of overweight and obesity in children. Data from the United States indicate that the rate of obesity was 13.9% for preschoolers and 18.4% for school-aged children in 2015–2016 [1]. The differing rates of obesity by age highlight the significance and necessity of identifying risk factors that influence childhood obesity in the early stages of life. A comprehensive understanding of these factors can contribute to the development of effective interventions.

Early childhood is a critical developmental stage for establishing health behaviors, including eating behaviors [2]. Compared to children with healthy eating behaviors, those with unhealthy eating behaviors are more likely to experience extra weight gain [3]. Some characteristics of children’s unhealthy eating behaviors associated with higher child body mass index (BMI) include higher responsiveness to food and emotional overeating [4, 5]. In contrast, healthy eating behaviors, such as a higher satiety response and slower eating, are considered protective factors against childhood obesity [5, 6]. Numerous factors influence children’s eating behaviors; notably, researchers have emphasized parents’ influence in establishing child eating behaviors [7, 8].

Parenting stress is a main factor that influences childhood obesity. Parenting stress is defined as an individual parent’s perception of stress regarding the parental role experienced as daily hassles related to a child’s behaviors [9]. In recent review studies, increased parenting stress was found to result in higher rates of childhood obesity and affect children’s health behaviors, including a child’s diet, physical activity, and sedentary behavior [10]. In a cross-sectional study, children in families with higher parenting stress due to economic hardship were more likely to consume foods high in saturated fats and added sugars [11]. Although there is evidence of an association between parenting stress and children’s health behaviors, the nature of the association is still not fully understood, possibly due to a lack of understanding of the mediating factors.

During the early developmental stages of life, children’s principal interactions are with their parents. Parents may guide their children through interactions with them [12]. High-quality interaction is often defined as mutuality and synchrony between parents and children [12]. The quality of the parent–child relationship gradually develops as parents and children adjust their behaviors [13]. In turn, the parent and child learn how to respond to each other’s behaviors and reactions. In a family with high-quality of relationship, the parent appropriately responds to the child’s behaviors and emotional expression (i.e., appropriate communication) [14]. Moreover, parents employ appropriate discipline techniques, including limit setting [15]. Parents who have a high-quality relationship with their children also enjoy their role as parents [16]. However, parents’ insecure attachment to the child has been associated with poor parenting practices, such as fewer mealtime routines, fewer limitations for watching television, and a greater use of unhealthy feeding practices, all of which can contribute to children’s unhealthy behaviors and obesity [17].

**Fig. 1:**
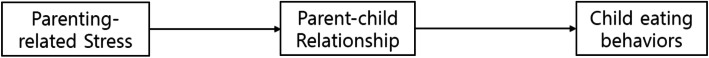
Theoretical Framework

According to the family systems theory, one family member is not independent from the others; rather, members continuously interact and influence each other and respond to events as a system [18]. Within the parent–child subsystem, parenting stress may influence the quality of the relationship between parents and children, which, in turn, may influence children’s health behaviors, including eating behaviors. Understanding the mediating effect of the parent–child relationship on the association between parenting stress and children’s eating behaviors is important to understand the key risk factors within a family that influence the obesogenic family environment (Fig. 1). This would lead to effective interventions for better health outcomes. Thus, the purpose of this study was to examine the mediating effect of the parent–child relationship on the association between parenting stress and children’s eating behaviors in families with young children aged four to six years.

## Methods

### Design and sample

We used a cross-sectional study design with a convenience sampling approach. We included mothers or female guardians from families with children aged four to six years who met the following conditions: 1) being a mother or female guardian; 2) being the primary caregiver; 3) being an adult who speaks and reads English; 4) being an adult who communicates with their child in English; 5) having a child who does not have any chronic conditions such as type 1 diabetes or any other endocrine diseases diagnosed by a healthcare provider that can affect his/her diet or BMI; and 6) having a child who does not take medication on a regular basis that influences his/her diet or BMI. Mothers of families with at least one eligible child participated in the study. If a mother had more than one eligible child, we asked the mother to select one child to provide information for the child. We recruited families from local communities in the Midwestern area of the United States (US), as well as from online communities in the US.

### Data collection

The institutional review boards of University of Wisconsin-Madison (#2018–0829) and Chungnam National University (201909-SB-170-01) reviewed and approved this study. We used multiple participant recruitment strategies, including direct contact, flyers, and snowball sampling, to promote the recruitment of participants. We collected data from November 2018 to July 2019. We selected local preschools (university-affiliated, private, and Head Start program preschools), local libraries, and community activity facilities such as the Young Men’s Christian Association (YMCA). We asked preschool teachers and administrators to place a flyer in the cubby of each child in their classrooms. Trained research staff and the coauthor visited the sites as needed to answer the questions of potential participants when they dropped off or picked up their children. We asked each participating family to introduce the study to other potentially eligible families. We also recruited participants by posting flyers online on social media, in local newsletters, and on online community websites such as Facebook, Craigslist, and blogs to expand our reach to additional potential participants.

We used a two-phase web-based survey developed in the Research Electronic Data Capture (REDCap) application hosted at the University of Wisconsin-Madison [19, 20]: 1) a screening survey to confirm the eligibility of the potential participants, and 2) the full survey with an accompanying informed consent form. Families who were interested in participating in the study identified an eligible child in their family. The mother of the eligible child accessed the initial survey through a publicly available survey link using a QR code or a website address, completed the initial online survey screening form, and submitted it to the research team. Once our research team reviewed the completed initial screening survey, we sent the full survey link to participants whose eligibility had been verified. After providing informed consent to participate in the study, the participants were able to access the questionnaires at their convenience for up to one month. The participants completed the survey within approximately 30 min. A research assistant sent each participant at least one weekly reminder email if the participant did not finish the survey within a week. If necessary, two weekly reminder emails were sent. Upon completion of the survey, each mother received a $30 e-gift card.

### Measures

#### Family and general characteristics

The families’ demographic data included the child’s age and gender, mother’s age, number of family members in the household, race/ethnicity, annual family income, and mother’s educational level. We asked the participants to report their children’s height and weight. To calculate the children’s BMI, we used the age- and sex-specific BMI percentiles provided by the US Centers for Disease Control and Prevention (CDC) [21]. We categorized the children’s BMI as “normal weight” or “overweight,” defined as a BMI in or above the 85th percentile based on the US population.

#### Parenting stress

The parenting stress of mothers was assessed using the Parenting Stress Index–Short Form (PSI-SF) [22]. The PSI-SF consists of 36 items rated on a 5-point Likert scale ranging from 1 (strongly disagree) to 5 (strongly agree). The PSI-SF contains three subscales: 1) personal distress, 2) parent–child dysfunction, and 3) child difficulty. Summing the items yields a total parenting stress score as well as sub-scale scores for parental distress, dysfunctional interaction, and child difficulty, with higher scores reflecting higher levels of parenting stress. The raw score could be converted into percentiles, with scores in the 16th to 84th considered normal, 85th to 89th considered high, and 90th percentile or higher considered clinically high [23]. We used the total score, which ranges from 36 to 180, to develop the model, with higher scores indicating higher levels of parenting stress. The instrument demonstrated excellent internal reliability (Cronbach’s alpha = .83) [22]. The Cronbach’s alpha for the study sample was .97.

#### Parent–child relationship

We assessed the participants’ parent–child relationship using the Parent–Child Relationship Inventory (PCRI), which measures parents’ self-reported parenting skills as well as their attitudes toward parenting and their children [24]. The instrument has 36 items which yields scores for the following eight subscales: 1) parental support (SUP), 2) satisfaction with parenting (SAT), 3) involvement (INV), 4) communication (COM), 5) limit setting (LIM), 6) autonomy (AUT), 7) role orientation (ROL), and 8) social desirability (SD). Higher scores indicate a higher quality parent–child relationship. The instrument demonstrated good reliability (Cronbach’s alpha = .79; test–retest correlation coefficient: .68) [24]. For this study, we selected the following PCRI subscales: SUP, SAT, INV, COM, and LIM. The overall Cronbach’s alpha for the study sample was .83. Cronbach’s alpha for the selected subscales ranged from .63 to .82; thus, SAT, COM, and LIM were chosen for model development, having showed Cronbach’s alphas of at least 0.70.

#### Children’s eating behaviors

Children’s eating behaviors were assessed using the Children’s Eating Behaviors Questionnaire (CEBQ) [25]. The CEBQ contains 35 items rated on a 5-point Likert scale ranging from 1 (never) to 5 (always). The instrument includes eight subscales: 1) food responsiveness (FR), 2) emotional overeating (EOE), 3) enjoyment of food (EF), 4) desire to drink (SD), 5) satiety responsiveness (SR), 6) slowness in eating (SE), 7) emotional undereating (EUE), and 8) fussiness (FF). For the study, we selected two unhealthy eating behaviors, namely, FR and EOE, for data analysis. The CEBQ demonstrated good reliability, with Cronbach’s alphas ranging from .72 to .91 among the subscales [25]. The Cronbach’s alphas for the study sample were 0.85 for FR and 0.86 for EOE.

### Analytic strategy

Data analysis was performed using SPSS Statistics for Windows (version 26.0; SPSS Inc., Chicago, IL, USA) and Mplus (version 8.4; Los Angeles, CA, USA). We generated descriptive statistics for each variable, including measures of frequency for categorical variables and mean and central tendency for continuous variables. With missing data analysis, we found that the majority of missing data met the missing completely at random (MCAR) assumption with one exception (i.e., COM), which was assumed to be missing at random (MAR) according to Little’s test [26]. We input the missing values through multiple imputations using chained equations [27]. We also ran a Pearson’s correlation to examine the bivariate associations between variables.

To address the study’s purpose, we used structural equation modeling to develop a path model with confirmatory modeling based on robust maximum likelihood, producing a model with direct, total, and indirect effects among variables. We used the fit indices of root mean square error of approximation (RMSEA), the comparative fit index (CFI), Tucker-Lewis index (TLI), and standardized root mean square residual (SRMR) to test the model fit [28]. An RMSEA value less than 0.05 indicates a good fit, while up to 0.1 represents a mediocre fit. Moreover, an RMSEA *p*-value of less than .05 is considered a good fit. CFI and TLI values equal to or greater than .9, and close to 1.0, suggest a good fit and an SRMR value less than .08 is considered also a good fit.

## Results

### Sample characteristics

The sample characteristics were listed in Table 1. The sample included 172 mothers of children aged four to six years. The mean age of the children was 4.92 (SD 0.89) years, and 50% of them were girls. Two–thirds (71.2%) of the children were non-Hispanic White, and 15.3% were Black. More than 50% of children had private health insurance. Less than half of the children (44.2%) were either overweight or obese. Among the mothers, 60.9% had a college education or higher, and 68% were employed in either full or part-time jobs. Regarding households, 84.3% of the families had at least two adults in the house, and more than half of the families (64.3%) earned less than $60,000 as their annual family income. Approximately 70% of mothers had a normal range of parenting stress, while 27.3% of mothers had clinically significant parenting stress.

**Table 1:** Sample characteristics (*n* = 172)

Category	Subcategory	Parents (*n* = 172)	Children (*n* = 172)
Age [mean (SD)]			4.92 (0.89)
Sex [n (%)]	Female	172 (100%)	86 (50%)
Birth order [n (%)]	1st		91 (52.9%)
	2nd or later		74 (43.1%)
Race [n (%)]	Non-Hispanic White		121 (71.2%)
	Black or AA		26 (15.3%)
	Other (Hispanics, Asians, American Indians, or multi-racial)		23 (13.5%)
Health insurance [n (%)]	Private		65 (38.2%)
	Self-purchased		30 (17.6%)
	Medicaid		64 (37.6%)
	No insurance		11 (6.5%)
Education [n (%)]	At least high school graduate	67 (39.2%)	
	At least college or university graduate	104 (60.9%)	
Employment [n (%)]	Full time or self employed	80 (47%)	
	Part-time	36 (21.2%)	
	Non employed	40 (23.5%)	
	Student	4 (5.9%)	
Number of adults in the household [n (%)]	1	27 (15.7%)	
	2 or more	140 (84.3%)	
Family income [n (%)]	Less than $19,999	14 (8.3%)	
	$20,000–$39,999	51 (30.4%)	
	$40,000–$59,999	43 (25.6%)	
	$60,000–$79,999	36 (21.4%)	
	$80,000–$99,999	8 (4.8%)	
	More than $100,000	16 (9.5%)	
Weight status [n (%)]	Normal weight		91 (55.8%)
	Overweight or obese		72 (44.2%)
Parenting stress [n (%)]	Normal	121 (70.3%)	
	High	4 (2.3%)	
	Clinically high	47 (27.3%)	

*SD* standard deviation, *AA* African-American

### Bivariate correlations between variables

The bivariate correlations between variables are presented in Table 2. Parenting stress was correlated with subcategories of the parent–child relationship (SAT [*r* = − .59, *p* < .01]; COM [*r* = .47, *p* < .01; LIM [*r* = − 0.81, *p* < .01]). The parent–child relationship subsets were correlated with children’s eating behaviors; SAT was inversely correlated with FR and EOE (*r* = −.21, *p* < .01; *r* = − .27, *p* < .01, respectively), and LIM was inversely correlated with FR and EOE (*r* = .37, *p* < .01; *r* = .44, *p* < .01, respectively). However, COM was not correlated with any of the children’s eating behaviors.

**Table 2:** Bivariate correlations between variables

	Parenting stress	SAT	COM	LIM	FR
SAT	−.59^**^	1			
COM	.47^**^	−.33^**^	1		
LIM	−.81^**^	.63^**^	−.39^**^	1	
FR	.30^**^	−.21^**^	−0.05	−.37^**^	1
EOE	.40^**^	−.27^**^	0.05	−.44^**^	.80^**^

*SAT* Satisfaction with Parenting, *COM* Communication, *LIM* Limit Setting, FR Food Responsiveness, *EOE* Emotional Overeating

* < .05 (2-tailed); ** < .01 (2-tailed)

### Associations among factors in the SEM model

The model fit indices, including RMSEA, CFI, TLI, and SRMR values, indicate a good model fit. The RMSEA of the model was 0.125, which was close to a mediocre fit, and the *p*-value of the RMSEA was 0.02, which was close to a good fit. The CFI value was .97, the TLI value was .91, and the SRMR value was .04, all considered good fits.

The path associations among these factors are listed in Table 3. Parenting stress was negatively associated with the parent–child relationship subcategories of SAT (b* = − 0.69, *p* < .01) and LIM (b* = − 0.82, *p* < .01) and positively associated with COM (b* = 0.45, *p* < .01). Regarding the association between the parent–child relationship subcategories and children’s unhealthy eating behaviors, two subcategories, COM and LIM, were negatively associated with FR (b* = − 0.24, *p* < .01; b* = − 0.46, *p* < .01, respectively). Moreover, LIM was also negatively associated with EOE (b* = − 0.49, *p* < .01).

**Table 3:** Structural Parameters of the relationships

Path	b	S.E.	*p*-value	95% CI^a^		b*
Direct effects						
Parenting stress ➔ SAT	−0.01	0.001	<.01	−0.009	− 0.007	− 0.69
Parenting stress ➔ COM	0.01	0.001	<.01	0.005	0.009	0.45
Parenting stress ➔ LIM	−0.02	0.001	<.01	−0.018	− 0.015	− 0.82
SAT ➔ FR	0.02	0.194	.95	−0.366	0.393	−0.01
COM ➔ FR	−0.46	0.150	<.01	−0.755	−0.167	− 0.24
LIM ➔ FR	−0.66	0.129	<.01	−0.913	−0.420	− 0.46
SAT ➔ EOE	−0.03	0.208	.91	−0.413	0.382	−0.01
COM ➔ EOE	−0.31	0.158	.05	−0.617	0.003	−0.15
LIM ➔ EOE	−0.73	0.146	<.01	−1.014	− 0.441	−0.49
Total effects						
Parenting stress ➔ … ➔ FR	0.008	0.001	.02	0.004	0.011	0.26
Parenting stress ➔ … ➔ EOE	0.010	0.002	<.01	0.007	0.014	0.34
Indirect effect (Mediating effect)						
Parenting stress➔SAT➔FR	0.000	0.002	.95	−0.003	0.003	−0.00
Parenting stress➔COM➔FR	−0.003	0.001	<.01	−0.005	−0.001	− 0.11
Parenting stress➔LIM➔FR	0.011	0.002	<.01	0.006	0.015	0.38
Parenting stress➔SAT➔EOE	0.000	0.002	.90	−0.003	0.004	0.01
Parenting stress➔COM➔EOE	−0.002	0.001	.06	−0.004	0.000	−0.07
Parenting stress➔LIM➔EOE	0.012	0.003	<.01	−0.007	0.017	0.40

*SAT* Satisfaction with Parenting, *COM* Communication, *LIM* Limit Setting, *FR* Food Responsiveness, *EOE* Emotional Overeating

^a^= 95% confidence interval; b = unstandardized estimates; b* = standardized estimate; *p*-value = 2-tailed p test

The total effect of parenting stress on children’s unhealthy eating behaviors was significant (b* = 0.26, *p* = .02; b* = 0.34, *p* < .01), allowing us to examine the mediating effects of the mediating factors (Table 3). The mediating effect of the parent–child relationship subcategories on the association between parenting stress and children’s unhealthy eating behaviors was presented as an indirect effect of the model. Communication mediated the association between parenting stress and food responsiveness (b* = − 0.11, *p* < .01). Furthermore, the mediating role of limit setting was shown in the association between parenting stress and food responsiveness (b* = 0.38, *p* < .01), as well as the association between parenting stress and emotional overeating (b* = 0.40, *p* < .01).

## Discussion

This study aimed to examine whether the parent–child relationship mediates the association between parenting stress and children’s eating behaviors. Parenting stress is known to be associated with children’s eating behaviors; however, the underlying mechanism of this association has not been clearly established [29]. To understand this complicated mechanism, prior studies have focused on exploring the mediating role of parental feeding practices [10]. We explored a possible mechanism by examining whether the quality of the relationship between parents and children mediates the association between parenting stress and children’s unhealthy eating behaviors. We used three subcategories of the parent–child relationship: parents’ satisfaction with parenting (i.e., satisfaction), their awareness in communication with their children (i.e., communication), and their perception of control regarding child discipline (i.e., limit setting). Communication mediated the association between parenting stress and children’s food responsiveness. Furthermore, there was a mediating role to limit setting in the association between parenting stress and children’s food responsiveness, as well as between parenting stress and children’s emotional overeating.

Our findings indicate that certain subcategories of the parent–child relationship (i.e., communication and limit setting) were negatively associated with unhealthy eating behaviors in children. According to Gerard [30], the quality of communication between parents and children represents how well parents respond to their children in various circumstances. Parents who have healthy communication with their children can be considered empathetic [31], and healthy communication plays a protective role in children’s engagement in risky behaviors [32]. Limit setting is considered a parenting discipline technique for establishing appropriate behaviors in children [33]. Children with parents who do not establish limits for them may feel insecure and are more likely to exhibit problematic behaviors [34]. We found that specific subcategories of the parent–child relationship, including quality of communication and limit setting, were negatively associated with children’s unhealthy eating behaviors.

Based on our findings, an association between parenting stress and children’s unhealthy eating behaviors may be driven by inappropriate parental awareness in communication with their children (i.e., communication) and inappropriate parental perception of control over the disciplining of their children (i.e., limit setting). In a systematic review study, it was found that parents whose children were overweight or obese were less likely to limit or control what their children eat (i.e., food items) or how (i.e., eating behaviors) [35]. This finding supports emerging evidence of a negative association between the quality of the parent–child relationship and childhood obesity [36]. In a longitudinal study, toddlers who had a poor quality relationship with their mothers were more likely to be overweight or obese later in their childhood [37].

We also found that parenting stress was associated with three subcategories of the parent–child relationship (i.e., satisfaction, communication, and limit setting). The negative association between parenting stress and the quality of the parent–child relationship subcategories may be driven by a complicated mechanism. The important point regarding parenting stress is that its source is not only the parents’ personal perception of stress related to parenting but also their relationship with their children, and parenting stress could depend on the children’s personal characteristics [38]. The relationship between a parent and a child is a dynamic interaction that drives both parents’ and children’s behaviors [39], and the quality of the interaction has been examined to better understand children’s social, behavioral, and cognitive development [40]. Thus, the close association between parenting stress and the parent–child relationship may be important for understanding the dynamics of the influence of parenting stress on children’s eating behaviors and obesity.

The influence of parenting stress on the parent–child relationship needs to be interpreted with caution. As previously mentioned, the parent–child relationship is not unidirectional; rather, it is bidirectional [41]. For example, in families with children with type 1 diabetes, communication and interaction between parents and children regarding management of the condition was found to be associated with parenting stress [42]. However, there is limited evidence supporting an association between parenting stress and the quality of the parent–child relationship in families of children with obesity. In this study, we did not assess children’s stress levels in terms of their relationship with their parents. It is possible that children are influenced by the stress level of their parents manifested in the quality of the parent–child relationship. In turn, children’s stress may influence their eating behaviors as a coping mechanism. Further research is needed to explore the association between children’s stress levels and eating behaviors.

To understand the mediating effect of the subcategories of the parent–child relationship on the association between parenting stress and children’s eating behaviors, we need to understand how humans respond to stress. A child’s eating behaviors may be a mechanism for coping with tension in a low-quality relationship with their parents [43]. Responses to stressors, which could be stressful events or events perceived as threatening by an individual, are often described as biological or behavioral. Biological responses to stress are more likely hormonal, involving the hypothalamic–pituitary–adrenal (HPA) axis to maintain homeostasis in the body [44]. Given the connection with biological responses, behavioral coping mechanisms for stress primarily draw attention to eating behaviors. Individuals with high stress levels may be more likely to respond to food and may tend to consume high–calorie foods [45]. Furthermore, individuals with high stress levels may experience more emotional eating and may seek high–fat and carbohydrate–dense foods [46].

Stress arises when an individual perceives the demands placed on them as exceeding their adaptive capacity. Stress can also involve the inability to handle or the challenge of dealing with events that are perceived as threatening [47]. A certain level of stress is considered adaptive; however, consistent and chronic exposure to stressors results in adverse health outcomes [48]. Based on the findings of this study, healthy communication between parents and their children may alleviate tension in the relationship between them, which may reduce children’s stress levels. Furthermore, the children of parents who set appropriate limits feel more secure and confident, and they can thus engage in healthy behaviors [49]. Therefore, the parent–child relationship, particularly parents’ communication and limit setting skills, may be an important intervention component for improving children’s unhealthy eating behaviors in families with high parenting stress levels. In a recent intervention study employing parents’ communication skills and parental monitoring during mealtime, it was found that the intervention was effective in increasing the frequency of family mealtime, increasing parental concerns about their children’s weight, and decreasing parental restriction to eat certain foods, all of which are considered healthy practices [50]. Thus, further development of intervention programs to strengthen the parent–child relationship would be beneficial to improving children’s eating behaviors.

This study has several limitations. First, due to the cross-sectional study design, there is no evidence to indicate a causal association between the variables in the SEM modeling. Moreover, because the study outcomes were drawn from mostly White, middle-income families, the results may not be applicable to families from other socioeconomic classes or from other racial or ethnic backgrounds. We also relied on parents’ self-reported information regarding their relationship with the child as well as on the child’s eating behaviors, thus a validated assessment of the study variables may be limited. Specifically, children’s BMI based on mothers’ self-reports of their child’s height and weight was inconsistent with the national average of overweight and obesity rates. In addition, we used an online survey method. Technology-based surveys may present an obstacle for some potential participating families. One-third of our sample comprised White families, and more than half of the mothers completed at least a high school education. Furthermore, we used subcategories of the parent–child relationship; thus, caution needs to be exercised in interpreting the mediating role of the association between parenting stress and children’s eating behaviors and obesity.

Despite these limitations, this study has important implications for future research. Subsequent studies to assess the key concepts addressed in this study can be undertaken. These complicated concepts can be approached through multiple assessment methods, such as the use of biological markers (e.g., salivary cortisol) to assess parents’ and children’s stress and to assess child obesity and other metabolic risk factors (e.g., BMI, waist circumference, and systolic blood pressure). Moreover, researchers may consider enhancing the rigor of the assessment of the parent–child relationship from the children’s perspective by employing multiple methodologies, such as observational measures. Further research would be conducted with a larger sample to target more racially or ethnically diverse participants with different educational backgrounds. Furthermore, in future studies, researchers would consider developing a study to assess the long-term influence of the parent–child relationship during preschool on health behaviors and obesity later in childhood. Finally, the development of an intervention study to evaluate the efficacy of stress management in the context of improving the parent–child relationship (i.e., parents’ communication and limit setting skills) to reduce childhood obesity is highly recommended.

## Conclusion

We examined the mediating effect of several subcategories of the parent–child relationship (i.e., satisfaction with parenting, communication, and limit setting) on the association between parenting stress and children’s eating behaviors and found that appropriate communication and limit setting between parents and children may be a mediating factor. Through this study, we contributed to a better understanding of the complicated relationship between parenting stress and children’s eating behaviors. Parenting stress was negatively associated with the quality of the relationship between parents and children. In turn, the quality of the parent–child relationship was almost significantly associated with children’s unhealthy eating behaviors. Therefore, the parent–child relationship could be an important intervention component for improving children’s eating behaviors in families with high levels of parenting stress.

## Data Availability

The datasets analyzed during the current study are available from the corresponding author upon reasonable request.

## Abbreviations

BMI: Body Mass Index
YMCA: Young Men’s Christian Association
REDCap: Research Electronic Data Capture
CDC: Centers for Disease Control and Prevention
PSI-SF: Parenting Stress Index–Short Form
PCRI: Parent–Child Relationship Inventory
SUP: Parental Support
SAT: Satisfaction with Parenting
INV: Involvement
COM: Communication
LIM: Limit Setting
AUT: Autonomy
ROL: Role Orientation
SD: Social Desirability
CEBQ: Children’s Eating Behaviors Questionnaire
FR: Food Responsiveness
EOE: Emotional Overeating
EF: Enjoyment of Food
SD: Desire to Drink
SR: Satiety Responsiveness
SE: Slowness in Eating
EUE: Emotional Under Eating
FF: Fussiness
MCAR: Missing Completely at Random
MAR: Missing at Random
RMSEA: Root Mean Square Error of Approximation
CFI: Comparative Fit Index
TLI: Tucker-Lewis Index
SRMR: Standardized Root Mean Square Residual
